# The genetic orchestra of salicylic acid in plant resilience to climate change induced abiotic stress: critical review

**DOI:** 10.1007/s44154-024-00160-2

**Published:** 2024-06-17

**Authors:** Mohamed Elsisi, Moaz Elshiekh, Nourine Sabry, Mark Aziz, Kotb attia, Faisal Islam, Jian Chen, Mohamed Abdelrahman

**Affiliations:** 1 School of Biotechnology, Nile University, Giza, 12588 Egypt; 2 College of Science, King Saud University, P.O. Box 2455, 11451 Riyadh, Saudi Arabia; 3 International Genome Center, Jiangsu University, Zhenjiang, 212013 China

**Keywords:** Mitigating abiotic stress, Climate change, Salicylic acid, Genetic engineering, CRISPR

## Abstract

Climate change, driven by human activities and natural processes, has led to critical alterations in varying patterns during cropping seasons and is a vital threat to global food security. The climate change impose several abiotic stresses on crop production systems. These abiotic stresses include extreme temperatures, drought, and salinity, which expose agricultural fields to more vulnerable conditions and lead to substantial crop yield and quality losses. Plant hormones, especially salicylic acid (SA), has crucial roles for plant resiliency under unfavorable environments. This review explores the genetics and molecular mechanisms underlying SA's role in mitigating abiotic stress-induced damage in plants. It also explores the SA biosynthesis pathways, and highlights the regulation of their products under several abiotic stresses. Various roles and possible modes of action of SA in mitigating abiotic stresses are discussed, along with unraveling the genetic mechanisms and genes involved in responses under stress conditions. Additionally, this review investigates molecular pathways and mechanisms through which SA exerts its protective effects, such as redox signaling, cross-talks with other plant hormones, and mitogen-activated protein kinase pathways. Moreover, the review discusses potentials of using genetic engineering approaches, such as CRISPR technology, for deciphering the roles of SA in enhancing plant resilience to climate change related abiotic stresses. This comprehensive analysis bridges the gap between genetics of SA role in response to climate change related stressors. Overall goal is to highlight SA's significance in safeguarding plants and by offering insights of SA hormone for sustainable agriculture under challenging environmental conditions.

## Introduction

Climate change refers to long-term alterations in weather patterns, predominantly attributed to the emission greenhouse gases originating from either natural processes or human activities (Mikhaylov et al. 2020; Fawzy et al. 2020; Skendžić et al. 2021; Shahid et al. 2021). Climate change events are considered the main threat to food security on a worldwide scale. Crop yields have decreased in many regions around the world as a consequence of climate change in the forms of increased drought and flooding events as well as extreme heat and cold waves (Agesa et al. 2019; Manabe 2019; Abdelrahman et al. 2021a)(Agesa et al. 2019; Manabe 2019; Abdelrahman et al. 2021a). It is predicted to influence total crop production by 9% and 23% in 2030s and 2050s, respectively, with huge differences across countries and crops (Haile et al. 2017. Recent estimates from the climate change predict increased water deficits and flash drought events by erratic rainfalls in the coming years (Wassmann et al. 2009; Mehana et al. 2021). Furthermore, drought intensity and frequencies are expected to increase (Haile et al. 2020). In 2022, Texas and Louisiana rice farmers in USA struggled with strong drought and intense heat issues and as a result some fields were left abandoned because farmers couldn’t keep water on their rice crop. This scenario may continue to happen as drought take serious hold during summer and Colorado river reservoirs have been depleted. According to adequate water risk atlas of the world resources institute (https://www.wri.org/insights/highest-water-stressed-countries), several more countries are already suffering from acute water scarcity such as Mexico, Pakistan, South Africa, and large parts of China and India as do most of the Near Middle East and North Africa countries.

Heat, cold, and drought are considered as the main abiotic stresses driven by climate change. Other abiotic stresses that happen as a consequence of the main climate change stressors includes increased soil salinity. These stressors are increasingly affecting crop productivity and threatening global food security with an estimated crop yield loss of 42% for the eight most frequently produced crops worldwide (Fahad et al. 2017; Wani et al. 2017). These stressors are continually affecting plants, and if they are neglected, they can trigger slow development and growth or even death, which could result in significant crop loss (Nazar et al. 2017; Wani et al. 2017). The evolving climate brings about daily environmental fluctuations that notably affect the molecular, cellular, physiological, and morphological aspects of plant vegetative growth which have profound implications for the sustainability of agriculture(Mehana et al. 2021; Baidya et al. 2022). Furthermore, it captivates global temperature rising, warming the ocean, decreasing snow covered area and rising of sea level. Due to this global warming, the heat waves is increasing in magnitude and occurrence frequency. As a consequence, it increases those abiotic stresses on a world scale, especially with the unevenness and unpredictable manner (Eckardt et al. 2023). Plants evoke different plant hormones as chemical messengers that act as signals for activating mechanisms that help the plants to survive during the stress events. (Raza et al. 2019; Malhi et al. 2021; Imran et al. 2021). Plant hormones, known as phytohormones, promote plant growth, and enable plants to perceive and respond to unfavorable environmental stimuli. Basically, those plant hormones modulate physiological and molecular responses of plants for its survival under abiotic stresses. Moreover, they function either at their site of production or elsewhere in plants based on their transport (Kundu et al. 2022). Among these plant hormones, cytokinin (CKs), abscisic acid (ABA), ethylene (ET), jasmonic acid (JA) and salicylic acid (SA) have been recognized to play a critical role in response to abiotic stresses (Zhao et al. 2021a; Mukherjee et al. 2022; EL Sabagh et al. 2022). SA reported to be involved in different defense responses in addition to its importance in regulation of plant growth and development as well as responses to abiotic stresses. Salicylic acid (SA, C_7_H_6_O_3_) is an endogenous phytohormone that has caught researcher's attention in recent years. It is 2-hydroxy benzoic acid, a phenolic derivative that was originally considered an insignificant byproduct named after the Latin name of the willow tree (Salix). It was found to be an important endogenously produced signaling molecule capable of defending plants against both biotic and abiotic stressors. SA has been linked to a variety of functions, including plant development, growth, and stress responses (Ahmad et al. 2019; Saleem et al. 2021; Khan et al. 2022; Boamah et al. 2023; Yang et al. 2023). Several internal physiological activities, such as photosynthesis, nitrogen metabolism, proline (Pro) metabolism, and glycine betaine (GB) production, are regulated by SA (Khan et al. 2015; Shemi et al. 2021). Furthermore, it involves complex signaling pathways that activate defense-related genes and proteins that lead to multiple events that respond to and resist stress (Kumar 2014).

SA is synthesized through phenylalanine ammonia-lyase (PAL) and isochorismate (ICS) pathways. Both pathways derive from chorismic acid, a byproduct of the shikimate pathway (Lefevere et al. 2020; Lone et al. 2020; Li et al. 2022a). The ICS pathway operates in stressed chloroplasts and involves chorismate conversion to isochorismate by isochorismate synthase 1 (*ICS1)*, forming SA through amino acid conjugation. While the PAL pathway, is using phenylalanine, produces smaller SA amounts. The ICS pathway involves PAL and AIM1 are employed in this pathway to convert phenylalanine to SA via trans-cinnamic acid (tCA) and benzoic acid (BA) (Murphy et al. 2020; Sharma et al. 2020; Tyagi et al. 2022).

Climate-induced stresses regulate SA biosynthesis enzymes in stressed plants to act as a signaling molecule (Yang et al. 2023). SA mediates plant stress responses by regulating genes, enzymes, and molecules. SA receptors, *nonexpresser of pathogenesis-related genes,* NPR1 and NPR3/NPR4 activate or repress defense gene expression. NPR1 interacts with SA, influencing gene transcription and defense responses (Maier et al. 2011; Backer et al. 2019; Liu et al. 2020b). Although its mechanism is not fully understood, SA is a key regulator of physiological and biochemical processes throughout the plant's life cycle. It is essential for the regulation of many important plant processes, from photosynthesis to senescence (Zhang et al. 2023). For that reason, SA may be regarded as a ‘green guardian’ to plants, serving to protect plants from abiotic or biotic stressors by inducing their resistance and alleviating the effects of the stress, as well as regulating vital plant processes.

In recent years, significant discoveries have shed light on the importance of SA in enhancing plant resistance to biotic and climate change driven abiotic stresses. The role of SA in resistance to pathogen stresses has been extensively reviewed elsewhere (Benjamin et al. 2022). Several studies have been conducted on the biosynthesis pathway of SA in plants and its importance to abiotic stress resistance. However, some gaps are not yet fully understood in the mechanisms of SA in controlling abiotic tolerance in plants under the different scenarios of climate change. This review aims to emphasize the role of SA in abiotic stress resistance. We also discuss possible mechanisms by which SA employs its role in mitigating abiotic stresses. Further, we explored updates on the effect of SA on the different abiotic stresses in plants. This review article collects and describes recent published information to better understand the role of the SA crosstalk’s with other hormone signal system in plant stress resilience.

## Biosynthesis of SA in plants

**Fig. 1:**
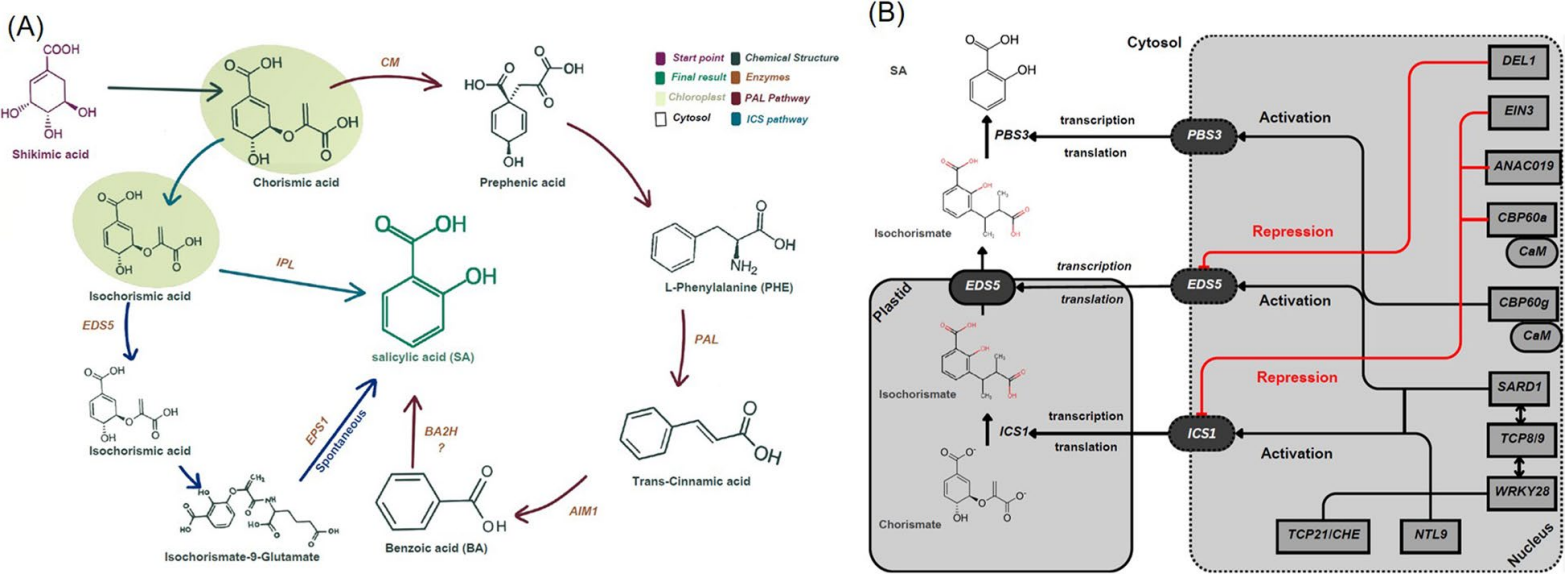
**A** Pathways of Salicylic Acid Biosynthesis in Plant, (**B**) Transcriptional Regulation network of Salicylic Acid Biosynthesis

Plants biosynthesize SA through two distinct pathways: the isochorismate (ICS) pathway and the phenylalanine ammonia-lyase (PAL) pathway (Fig. 1A). The ICS pathway takes place in the plastids while the PAL pathway takes place in the cytosol. Both pathways rely on chorismate, which is a primary metabolite and the end-product of the shikimate pathway (Hao et al. 2018; Mishra and Baek 2021; Zhang et al. 2021b). It is produced within the plastid, specifically in the chloroplast, through a seven-step enzymatic process of the shikimate pathway. This compound is synthesized from the combination of phosphoenolpyruvate (PEP) and erythrose-4-phosphate (E4P) (Ding and Ding 2020). The ICS pathway is specific to Brassicaceae family genome such as in Arabidopsis, whereas the PAL pathway is more specific in many other trees and crops such as rice (Yuan et al. 2009; Holland et al. 2019; Torrens-Spence et al. 2019; Wu et al. 2023). It is also possible, as in the case of soybeans, for both pathways to contribute equally. Additionally, there may even be variations in the regulation of SA production within a single plant (Lefevere et al. 2020). However, not all SA biosynthesis-involved enzymes have been identified in plants.

### ICS Pathway regulating SA biosynthesis

Genetic analysis in conjunction with biochemical techniques has paved the way for identifying and understanding the distinct stages involved in the biosynthesis of SA. Three main genes, *enhanced disease resistance5 EDS5*, *ICS1*, and *PBS3* were recognized as encoding three enzymes that establish the fundamentals of the ICS pathway of SA biosynthesis. Chloroplastic *AtICS1* (*SA induction deficient 2*, *SID2*) was identified by targeting plants with SA induction deficiency (Wildermuth et al. 2001). *ICS1* and its closely related homolog *ICS2* share homology with *PchA*, an isochorismate synthase (ICS) pivotal for the initial SA biosynthesis step in *Pseudomonas aeruginosa*. Both *ICS1* and *ICS2* play essential roles in SA biosynthesis, localizing within chloroplasts and catalyzing chorismate to isochorismate (IC) conversion in most plants (Wildermuth et al. 2001; Garcion et al. 2008a). While the *OsICS* gene's role in SA biosynthesis is less established, and alternative pathways might contribute to SA production (Table 1).

**Table 1:** Genes involved in the biosynthesis of salicylic acid

Gene	Organism	Function	References
*AtAIM1*	*Arabidopsis thaliana*	Beta-oxidation multifunctional protein, conversion of trans-cinnamic acid to benzoic acid	(Richmond and Bleecker 1999; Bussell et al. 2014)
*OsAIM1*	*Oryza sativa*	Beta-oxidation multifunctional protein, conversion of trans-cinnamic acid to benzoic acid	(Xu et al. 2023)
*AtPAL*	*Arabidopsis thaliana*	Phenylalanine ammonia-lyase, conversion of phenylalanine to trans-cinnamic acid	(Huang et al. 2010a)
*OsPAL*	*Oryza sativa*	Phenylalanine ammonia-lyase, conversion of phenylalanine to trans-cinnamic acid	(Tonnessen et al. 2015)
*AtICS*	*Arabidopsis thaliana*	Isochorismate synthase, conversion of chorismate to isochorismate	(Garcion et al. 2008b)
*OsICS*	*Oryza sativa*	Isochorismate synthase, conversion of chorismate to isochorismate	(Yokoo et al. 2018)
*PBS3*	*Arabidopsis thaliana*	Glutamate amidotransferase, conjugation of glutamate to isochorismate to produce isochorismate-9-glutamate	(Rekhter et al. 2019)
*EDS5*	*Arabidopsis thaliana*	Isochorismate transporter transports isochorismate from chloroplast to cytosol	(Rekhter et al. 2019)
*EPS1*	*Arabidopsis thaliana*	Enhanced pseudomonas susceptibility 1, conversion of isochorismate-9-glutamate to salicylic acid and 2-hydroxy-acryloyl-N-glutamate	(Zheng et al. 2009)

The *pbs3* mutant Arabidopsis plants displayed low SA, SAG levels and increased susceptibility to avirulent *Pseudomonas* strains and virulent *P. syringae* strains, with *PBS3* identified as a glycoside hydrolase 3 (GH3) protein family member and later confirmed as an isochorismate lyase (IPL) involved in SA biosynthesis (Li et al. 2023b). This function differs from bacterial PchB, with *PBS3* exhibiting interaction with SA. *PBS3* as an isochorismoyl-glutamate synthase (IGS), catalyzing IC adenylation and conjugation with Glu to yield isochorismoyl-9-glutamate (IC-9-Glu), which breaks down into SA and other compounds (Rekhter et al. 2019). Additionally, *enhanced pseudomonas susceptibility* 1 (*EPS1*), an enzyme enhancing SA production, was identified as an IC-9-Glu pyruvoyl-glutamate lyase (IPGL), indicating a distinctive SA biosynthesis pathway in plants like Arabidopsis compared to bacteria and other organisms (Chen et al. 2020).

In the bacterial system, the ICS pathway starts by converting chorismate to IC by the enzyme ICS. In Pseudomonas species, the *PmsCEAB* gene cluster, particularly the *PmsC* and *PmsB* genes, is vital for this pathway. *PmsC* shares sequence similarities with *E. coli*'s ICS and catalyzes chorismate to IC conversion. *PmsB* encodes an IPL responsible for IC to SA conversion in a two-step process (Mercado-Blanco et al. 2001; Cosima et al. 2003). Interestingly, the number of ICS homologs in plant genomes is limited, with the primary enzymatic steps occurring in the chloroplast. Recent research has demonstrated variations in the SA synthesis pathway in Arabidopsis compared to bacteria. This involves amino acid conjugation of IC, catalyzed by the *PBS3* gene, leading to the formation of SA. The ICS pathway significantly contributes to SA accumulation in Arabidopsis upon pathogen attack (Yang et al. 2015).

SA has two inactive vacuolar storage forms, SA-β-D-Glucose Ester (SGE) and SA-2-O-β-D-glucoside (SAG) The accumulation of SAG and SGE in vacuoles is substantial, and these forms can be converted into active usable forms through hydrolysis (Dean and Delaney 2008; Allasia et al. 2018; Zeier 2021).In response to pathogen attack, the overall level of SA (including SA + SAG/SGE) significantly rises, triggering the activation of the systemic acquired resistance (SAR)-dependent defense pathway. Furthermore, methylation of SA leads to the production of a volatile variant called methyl salicylate (MeSA), which can be produced by esterification of SA leading to increased membrane permeability (Hayat et al. 2010 ; Wani et al. 2017). MeSA is stored in the cell until a large amount of SA is needed, thus acting as an inactive precursor to SA (Hayat et al. 2010 ; Saleem et al. 2020).

### PAL Pathway regulating SA biosynthesis

In the PAL pathway, the synthesis of SA from phenylalanine (Phe) has been extensively explored. Several experiments demonstrated that plants can convert Phe into trans-cinnamic acid (tCA), a precursor for various phenolic compounds (Saunders and Olechno 1988; Rasmussen and Dixon 1999). This conversion is catalyzed by the enzyme phenylalanine ammonia-lyase (PAL), the initial enzyme in the phenylpropanoid pathway. Arabidopsis genome has four PAL genes, each encoding distinct enzyme variants (Huang et al. 2010b). PAL's role in SA production was confirmed through experiments reporting increased PAL expression accompanied by elevated SA levels during pathogen resistance. Suppression of PAL activity resulted in reduced pathogen-induced SA accumulation. Investigating the steps from tCA to BA revealed the possibility of SA synthesis through cinnamoyl-CoA β-oxidation, particularly in tobacco (Chong et al. 2001). Arabidopsis Aldehyde Oxidase 4 (AAO4) was identified as contributing to BA production in seeds, potentially acting as a precursor for SA synthesis (Ibdah et al. 2009). While PAL plays a part in SA biosynthesis, its functions extend beyond, affecting the production of various compounds potentially related to defense mechanisms. The conversion steps from Phe to SA are crucial, as prephenate can diverge into various biosynthetic routes PAL transforms Phe into tCA.

Moreover, the PAL pathway involves the multifunctional protein (MFP) family member Abnormal inflorescence meristem1 (*AIM1*), which was identified through mutant analysis in Arabidopsis. *AIM1*, present in both Arabidopsis and rice, participates in various metabolic processes, including fatty acid metabolism, hormone metabolism, and amino acid metabolism. *AIM1*'s involvement in converting tCA into BA suggests a role in the PAL pathway. This enzyme has diverse substrates due to its beta-oxidation function. Although knockout plants involving the *AIM1* gene complex are intricate to interpret, they remain valuable for studying SA biosynthesis and defense responses (Xu et al. 2017b; Jia et al. 2023). The hypothesis of a BA 2-hydroxylase (BA2H) enzyme converting BA to SA was suggested, supported by activity coinciding with SA buildup in tobacco (Staswick et al. 1995; Sawada et al. 2006; Steinwand 2017). However, the gene responsible for BA2H has not been definitively identified. To gain comprehensive insights into plant SA biosynthesis, further studies are required.

## Regulation of salicylic acid biosynthesis

Mechanisms regulate SA concentration to maintain optimal levels is essential for plant function. Genes involved in SA biosynthesis are tightly controlled (Fig. 1b). Most of these genes are induced in response to stress, and SA accumulation can further stimulate its biosynthesis through transcriptional regulation. Phenomena like post-transcriptional regulation also impact SA biosynthesis which is a cornerstone of SA-mediated plant immunity (Zhong et al. 2021).

### Positive transcriptional regulation

Positive transcriptional regulations are required to switch on the production of SA. Plants *Calmodulin-binding TFs*, such as *CaM-Binding Protein 60 g (CBP60g)* and *Systemic Acquired Resistance Deficient 1 (SARD1)*, facilitate pathogen-induced SA synthesis by regulating the transcription of the key SA biosynthesis genes like *ICS1* and *EDS5* (Pokotylo et al. 2019; Huang et al. 2020; Santisree et al. 2020). *WRKY* TFs like *WRKY28* and *WRKY46* are involved in regulating *ICS1* expression and *PBS3* (van Verk et al. 2011; Chen et al. 2021). Furthermore, *Teosinte branched1/cycloidea/proliferating cell factor (TCP)* members *TCP21, TCP8/TCP9, and NAC TF-like 9 (NTL9)* have also been identified as activators of *ICS1* during immune responses. *AIM1*, belonging to the MFP family, contributes to the conversion of tCA to BA in the PAL pathway. Also, the activation of SA biosynthesis is crucially influenced by calcium (Ca^2+^) signaling, which modulates the activities of *CBP60g* and the Ca2^+^/calmodulin-binding transcription factor *CAMTA3* (Zhou and Zhang 2020; Peng et al. 2021; Yuan et al. 2021).

### Negative transcriptional regulation

*CBP60a*, a homolog of *CBP60g*, negatively regulates *ICS1* expression upon CaM-binding, suggesting a complex interplay between different factors. NAC TFs, including *ANAC019/055/072*, have been identified as negative regulators of SA biosynthesis (Bian et al. 2021). The abscisic acid-responsive NAC019 (*ANAC019*) directly binds to *BA/SA carboxyl methyltransferase 1 (BSMT1)* and *ICS1* promoters, repressing SA production. Another SA negative regulator is *DP-E2F-like1 (DEL1)* and *Ethylene Insensitive 3 (EIN3)*. *DEL1* suppresses *EDS5* expression, while *EIN3* mediates the negative regulation of *ICS1* expression (Chen et al. 2009; Eichmann and Schäfer 2015). Loss of function *del1* mutant exhibits accumulated production of SA and increased disease resistance (Chandran et al. 2014).

## Possible mechanisms for the actions of SA in mitigating abiotic stress

As a consequence of climate change plants are frequently exposed to abiotic stressors; likely more than one at the same time. Most frequently high temperature together with water deficit and increased soil salinity are the most common scenario. The events of abiotic stresses increase the accumulation of endogenous SA in plants during stress (Chieb and Gachomo 2023). Exogenous application of SA was found to regulate and mediate numerous plant responses to abiotic stress which, although not extensively documented or understood, are promising ways to alleviate the damage done by these stressors.

### SA induces stomatal closure under stress conditions

SA's ability to promote stomatal closure was reported in experiments with SA exogenous treatment (Mori et al. 2001; Khokon et al. 2010; Panchal et al. 2016). Mutant Arabidopsis plants (carrying *constitutive expresser of pathogenesis-related genes-5 (cpr5*) and *accelerated cell death 6(acd6*)) exhibiting SA accumulation showed elevated drought tolerance and reduction in stomatal aperture (Miura et al. 2013). Furthermore, SA signaling in stomatal guard cells is mediated by its receptor NPR1 (Zeng and He 2010; Ding et al. 2018).

The SA signaling and its cross-talk with other signaling pathways in the guard cells are considered a cornerstone in conferring tolerance in plants. Recently, SA was reported to activate peroxidase-mediated reactive oxygen species (ROS) signal that is integrated into the Ca^2+^/CPK-dependent ABA signaling branch in the Arabidopsis guard cells. The model described by (Prodhan et al. 2018) explains the overlapping between both SA and ABA signaling in the guard cells (Fig. 3b). This mechanism is supported by the findings of Zhang et al. when conducted his studies on the *PtrWRKY75* gene which acts upstream of *PAL1* and promotes his expression by binding to its promoter under drought stress which promotes SA biosynthesis. SA increased ROS accumulation leading to smaller stomatal aperture (Zhang et al. 2020). Furthermore, the overexpressed *PtrWRKY75* lines showed improved water-use efficiency under drought stress by reducing their stomatal conductance and transpiration rate.

### SA regulation of osmolytes under stress conditions

Osmolytes are soluble organic compounds that play a role in regulating the amount of water in the cell via a process called osmoregulation (Saleem et al. 2021). Osmoregulation is a vital defense mechanism against various abiotic stressors including salinity, heavy metal toxicity, and cold stress (Saleem et al. 2020; Sharma et al. 2020). Osmolytes, such as proline and GB, accumulate in the plant to alleviate stress by reducing the number of ROS and maintaining cell turgor. It has been known that SA controls osmolytes as a metabolic process involved in stress signaling. Exogenous SA has been found to interact with osmolytes and induce their accumulation in plants to alleviate stress (Szegediensis et al. 2005; Sharma et al. 2019). This SA based modulation mainly through enhancing the expression pattern of key biosynthetic genes(La et al. 2019a; Singh et al. 2022a).

### SA regulates proline production under stressful conditions

Proline is an amino acid that accumulates under stressful conditions and alleviates stress via many mechanisms including induction of stress-related enzymes, maintenance of membrane integrity, and chelation of heavy metals (Hayat et al. 2012; Saleem et al. 2021). Studies have shown that SA can increase the production of proline in plants under abiotic stresses, such as drought, salinity, and heat stress (Misra and Saxena 2009; Iqbal et al. 2014). Additionally, (Dawood et al. 2022) found that exogenous application of SA led to a significant increase in proline content in fava beans under simultaneous drought and salinity stress. It was proven that exogenous application of SA upregulates proline biosynthesis-related genes, e.g., *P5CSA* and *P5CSB* (*pyrroline-5-carboxylate synthase A* and *B*) while downregulating *PDH* (*proline dehydrogenase*) in drought stressed *Brassica rapa* (La et al. 2019b). At the same time, exogenous proline application was previously shown to induce SA production (mediated by NDR1-dependent signaling pathway) and was shown to modulate calcium (Ca^2+^)-mediated oxidative burst defense response in plants (Chen et al. 2001; La et al. 2019a).

### SA induces the synthesis of glycine betaine (GB) under stressful conditions

**Fig. 2:**
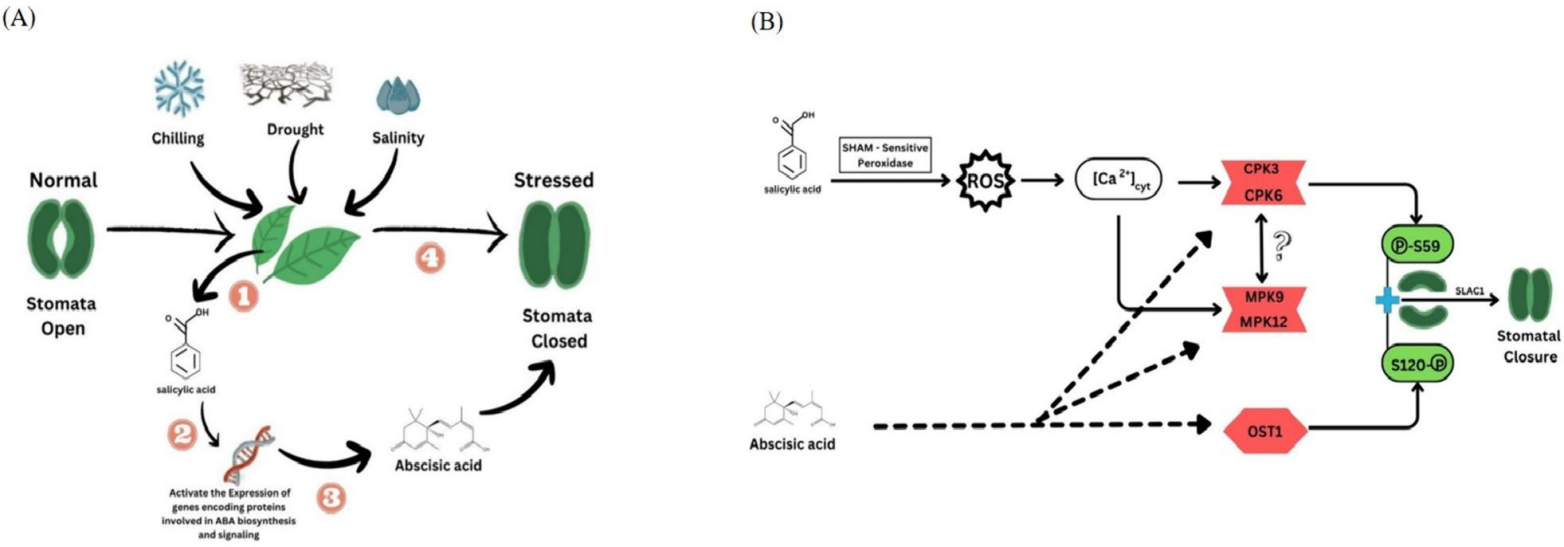
SA signaling and its cross-talk with ABA. **A** SA was found to increase the amount of ABA in response to stressors such as chilling, drought, and salinity which in turn has led to stomatal closure. **B** Shows Salicylic acid (SA) signaling in guard cells integrated with abscisic acid (ABA) signaling via the calcium-dependent protein kinase (CPK) pathway. SA triggers a ROS signal that activates CPKs, which then phosphorylate S59 and S120 of SLAC1, a protein that regulates stomatal closure. ABA also requires CPKs for the activation of SLAC1, but it also requires OST1, a protein kinase that is not involved in SA signaling. The two MAPKs, MPK9 and MPK12, also function downstream of ROS and Ca^2+^ in guard cell SA signaling and regulate SLAC1 activity indirectly. The molecular mechanism underlying the interdependence of the CPK-dependent and MPK-dependent pathways for SLAC1 activation is unknown

GB is a quaternary ammonium compound that helps improve plant growth, reduce osmotic stress, and regulate nutrient uptake under stress (Ali et al. 2020; Sharma et al. 2020; Singh et al. 2022b; Shohani et al. 2023). GB helps to protect plants from stress by adjusting cell osmotic balance, which helps to prevent cells from shrinking or bursting (Raza et al. 2023), stabilizing membrane integrity, which helps to prevent the leakage of cellular contents (Khan et al. 2014), protecting Rubisco activity, which is the enzyme that converts carbon dioxide into sugar (Bharwana et al. 2013), detoxifying toxic ions (Jagendorf and Takabe 2001). SA has been reported numerous times to induce the synthesis of GB, thus enhancing plant growth under various stressors including cold stress, heat stress, and drought stress (Khan et al. 2015; Sharma et al. 2020). SA can induce GB accumulation in the range of 0.5–2.5 mM, which is sufficient to protect plants from stress (Jagendorf and Takabe 2001). The increase in GB level mediated by SA can improve overall plant growth (Misra and Misra 2012). (Khan et al. 2014) showed that the alleviation of salinity-inhibited photosynthesis and growth by SA in mungbean involves GB. SA can induce GB accumulation by increasing methionine content and suppressing ethylene formation. A similar effect was observed with the application of SA-analog, 2, 6, dichloro-isonicotinic acid, on GB accumulation and alleviation of salinity-induced adverse effects on photosynthesis and growth. More recently, Abbaspour and Ehsanpour (Abbaspour and Ehsanpour 2020) reported GB accumulation in response to exogenous SA treatment, this increase was not accompanied with an increase in BADH (GB processing enzyme) activity. Further studies are needed to reveal the mechanism of which GB is regulated by SA (Fig. 2).

### SA regulated mineral uptake to elevate plant growth under stress

Plants require minerals to grow and survive. While abiotic stressors disrupt mineral uptake and lead to stunted growth and development. The mineral nutrient status of plants can affect their ability to tolerate stress. SA has been shown to regulate mineral uptake and metabolism in plants under stress. In addition, it has been shown to protect plant membranes and regulate the uptake (Alpaslan and Gunes 2001; Gunes et al. 2007). SA has been shown to improve photosynthesis in salt-stressed plants by decreasing cellular sodium (Na^+^) and chloride (Cl^−^) ions and increasing the content of nutrients. For example, SA supplementation strongly inhibited Na^+^ and Cl^−^ accumulation but stimulated nitrogen, iron, and copper concentrations in salt-stressed corn (Gunes et al. 2007). However, the mechanism that SA uses to selectively promote the beneficial elements uptake while hampering the toxic ones is not yet fully understood (Kaya et al. 2023). Furthermore, SA priming improved plant growth under different abiotic stress conditions by improving water, secondary metabolite and nutrient uptake. Those abiotic stresses such as salt (Gondor et al. 2023), mainly through controlling the root-shoot system mineral nutrients by adapting the influx of Na^+^ and K^+^ ions. Another example was explained by improved grain filling and grain protein content in high temperature stresses *Tritcum aestivum* (Ihsan et al. 2022). and *Cucumis Sativus* (Basirat and Mousavi 2022) treated by SA Moreover, SA treatment increased nitrogen uptake in mustard (*Brassica juncea*) plants grown in water deficit with low nitrogen supply conditions (Iqbal et al. 2022).

### SA induces major beneficial secondary metabolites in stressed plants

Secondary metabolites have been established as defense chemicals for plants under abiotic stress. There is extensive evidence that SA can directly and indirectly induce the synthesis of secondary metabolites in plants (Austen et al. 2019). A study reported that the exogenous application of SA to UV-B-exposed wheat plants increased the accumulation of anthocyanin and tocopherol, and also modulated the expression of pathogenesis-related (PR) proteins (Horváth et al. 2007a) Furthermore, the supplementation of SA in *Capsicum chinense* elicited the activity of PAL and caused subsequent increased vanillin production (Rodas-Junco et al. 2013). Numerous other studies have also reported an increase in PAL activity after the exogenous application of SA on plants including saffron (Tajik et al. 2019) and sage (Ejtahed et al. 2015); this led to an increase in the number of phenolic compounds in the plant. In another study by (Wen et al. 2008), it was shown that SA-mediated improved thermo-tolerance in grapes was a result of SA-induced accumulation of PAL mRNA, the synthesis of new PAL protein, and significant accumulation of phenolics.

SA application enhanced triterpenoid content in *Centella asiatica* through overexpression of triterpenoid biosynthesis-related genes, which led to enhanced anti-inflammatory activity (Buraphaka and Putalun 2020). In ginseng, SA induced accumulation of farensol, isochiapin B sesquiterpenoids, champhor, and cineole monoterpenoids by up-regulating genes corresponding to the enzymes involved in their syntheses such as farnesyl diphosphate synthase and isopentenyl pyrophosphate isomerise (Rahimi et al. 2014). In goji berries, the expression of *chalcone isomerase* gene and the total content of flavonoids were upregulated by exogenous SA treatment (Guan et al. 2014).

### SA controls responsive genes involved in stress resistance

SA induces SAR, which generates signaling responses to abiotic stress. These signals stimulate the expression of stress-defensive genes. Defensive genes aid plants in alleviating abiotic stresses via eliciting internal physiological and molecular responses. Recently, stress-responsive genes; SOS1 (Salt overly sensitive 1) and NA^+^/H^+^ exchanger (NHX1) were reported to be significantly upregulated in SA seed-primed pea plants exposed to salt stress (Ahmad et al. 2022). Furthermore, SA was reported to influence heat shock proteins (HSPs), such as HSP70 and Hsp17.6 (Sangwan et al. 2022). HSPs act as molecular chaperons that repair denatured proteins and stabilize the cell proteins against heat stress damage (Mittler et al. 2012). Furthermore, SA pretreatment regulated the transcription of the antioxidant enzymes genes *catalase* (*CAT*) and *ascorbate peroxidase* (*APX*), *APX,* and *GPX* in barely any plants under Cd-induced oxidative stress. Lower APX activity coupled with higher sensitivity to drought stress, salt stress, or cold stresses were reported in rice plants with *Osapx2* mutations. While plants with *OsAPX2* overexpression showed higher APX activity and stress tolerance were reported (Zhang et al. 2013). The elevation in the expression level of *Pathogenesis-related* (*PR*) genes and the endogenous levels of salicylic acid (SA) coincide during the activation of disease resistance in plants. Studies have shown that these genes are regulated by abiotic stresses and SA, suggesting that they serve a specific function during abiotic stress (Miura et al. 2013). In rice, the PR genes *PR4c* and *PR4d* were induced by SA and improved drought tolerance (Wang et al. 2011). The transcriptional coregulator *NPR1* is a key gene in the activation of SA-dependent stress defense genes. It was reported that SA-based oxidative and salt stress tolerance happens through NPR1- dependent pathway (Pan et al. 2006; Jayakannan et al. 2015). SA is also involved in several in upregulation of genes of several signaling pathways such as ABA and Jasmonic Acid (JA). Wang et al. reported an elevated expression of *NCED1* and *NCED2*, two important genes involved in ABA synthesis, followed by a subsequent increase in endogenous ABA concentration after exogenous SA treatment (Wang et al. 2018b). ABA accumulation appears to be a signaling process that follows SA sensing. The expression of the ABA biosynthesis genes, *SlZEP1*, *SlNCED1*, *SlAO1*, and *SlAO2*, was increased following SA treatment in salt-stressed tomato roots (Szepesi et al. 2009).

Furthermore, SA has a direct role in activating several transcription factors which involved in enhancing plant tolerance to abiotic stresses (Sakuma et al. 2006). In *Arabidopsis thaliana*, *DREB2A*, a transcription factor with a negative regulatory domain in its middle region, plays an important role in heat stress response. Deletion of the negative regulatory domain in *DREB2A* transforms it into a constitutively active form (*DREB2A CA*). Overexpression of *DREB2A CA* in transgenic Arabidopsis enhanced tolerance to heat/drought stress. The relationship between *DREB2A* and SA is complex and not fully understood. However, they work together to promote plant stress tolerance. Proteomic analysis investigations revealed that some transcription factors are differentially regulated via SA under abiotic stresses such as 14–3-3, NAC domain-containing proteins, and SET domain proteins (Kang et al. 2012).

### SA regulating redox signaling and antioxidant defense system

SA plays a fundamental role in regulating redox homeostasis and regulating the antioxidant defense system. This redox regulation is happen to occur by enhancing expression of redox regulating genes (such as, *TRXh5* and GRXC9) coupled with enhancing NPR1- dependent signaling pathway (La et al. 2019a). This redox modulation through transcriptional regulation enhanced abiotic stress tolerance, such as drought (La et al. 2019a). For instance, SA treatment on young grape leaves reduced the levels of thiobarbituric acid reactive substances (TBARS) and relative electrolyte leakage and increased the activity of antioxidant enzymes (Wang and Li 2006)​. Furthermore, SA contributed to cellular redox homeostasis through upregulating proline biosynthesis under drought stress conditions as well as the redox status including glutathione cycle enzymes and the antioxidant defense(La et al. 2019b). The antioxidant defense system comprises a complex network of genes and proteins that collectively orchestrate the production and elimination of ROS which acts as oxidant molecules under abiotic stress. These ROS, such as superoxide radicals (${O}_{2}^{-}$) and hydrogen peroxide (H_2_O_2_H_2_O_2_), possess high reactivity and can disrupt a wide array of biochemical metabolic pathways, including enzyme functions, DNA integrity, membrane permeability, and protein synthesis. Typically, plant cells have mechanisms in place to regulate ROS levels, ensuring they remain within manageable limits (Swanson and Gilroy 2010; Mhamdi and Van Breusegem 2018; Mansoor et al. 2022). In plant cells, this system involves approximately 152 genes that contribute to the synthesis of enzymes and non-enzymatic compounds responsible for both generating and eliminating ROS (Hideg et al. 2013; Dumanović et al. 2021).

ROS primarily act as metabolic by-products stemming from oxygen utilization, representing a potential source of cellular toxicity. Also, ROS are utilized as key signaling molecules in various physiological processes. These include growth and development, hormone signaling, cell apoptosis, cell cycle regulation, as well as responses to both biotic and abiotic stressors. This dual role of ROS highlights their significance as molecular messengers in plant biology (Miller et al. 2008; Nadarajah 2020; Sachdev et al. 2021).

Since H_2_O_2_ holds a vital role in plants' defense against abiotic stressors, the SA-induced H_2_O_2_ surge can potentially enable plants to withstand subsequent abiotic stressors as well (Faizan et al. 2021). This has been particularly evident in experiments involving exposure to heavy metals when rice roots were treated with SA before exposure to Cd stress, it initially increased the presence of H_2_O_2_, alongside higher levels of antioxidant molecules and enzyme activities. This enhanced antioxidant system effectively minimized the oxidative damage caused by Cd stress (Guo et al. 2007, 2009).

**Fig. 3:**
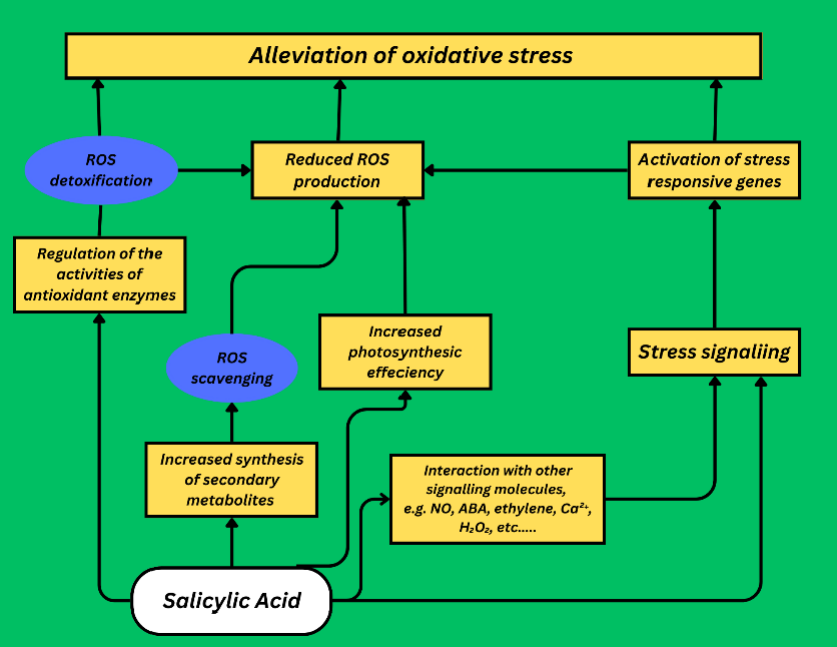
Some potential mechanisms in plants for oxidative stress tolerance carried out by salicylic acid

ROS, including some genes play a critical role in plants' responses to abiotic stresses. Transcription factors like *FtbZIP5* and *GarWRKY5* may enhance drought and salt tolerance through ROS-related pathways, potentially involving ROS scavenging genes, JA and SA-related genes (Guo et al. 2019; Gechev and Petrov 2020). The relationship between SA and ROS-scavenging enzymes like catalases (CATs) and ascorbate peroxidase (APX) is complex. CATs are highly efficient enzymes that rapidly convert H_2_O_2_ into water and oxygen (Azarabadi et al. 2017). Application of SA inhibited CAT activity, leading to a self-amplifying loop of ROS production. This, in turn, triggers the expression of defense-related proteins like *PR1* and culminates in SAR (Khokon et al. 2011; Mejía-Teniente et al. 2013). Similarly, APX, which is an enzyme with a strong affinity for H_2_O_2_, can also be influenced by SA. Supplementation with SA can enhance APX activity, aiding in the detoxification of ROS. This is particularly evident under conditions of stress, such as exposure to heavy metals or waterlogging (Fig. 3) (Torun 2019).

Furthermore, the antioxidant molecules ascorbate peroxidase (ASA), glutathione (GSH), malondialdehyde (MDA) and Glutathione persulfide (GSSH) play critical roles in mitigating oxidative damage caused by abiotic stresses. GSSH is involved in redox signaling through its association with H_2_S signaling pathways, ASA plays a role in maintaining redox balance by reducing H_2_O_2_, and MDA serves as an indicator of oxidative damage in cells. Genetic studies have indicated a linkage between GSH biosynthesis and endogenous SA signaling. SA levels have been correlated with changes in GSH levels in mutants deficient in catalase activity (Herrera-Vásquez et al. 2015; Chaouch et al. 2010). Several enzymes involved in GSH metabolism, such as glutathione synthetase (GSHS), glutathione reductase (GR), glutathione S-transferases (GST), and glutathione peroxidase (GPX), also show relationships with SA signaling (Njålsson and Norgren 2005; Nazar et al. 2011; Mostofa et al. 2015).

However, while the positive impacts of SA signaling have been extensively explored, some studies have yielded conflicting results. For instance, while SA-induced H_2_O_2_ production can be beneficial, it has also been associated with potentially detrimental outcomes, such as oxidative damage (Choudhury and Kumar Panda 2004; Hayat et al. 2010). Similarly, the effects of SA mutants on ROS responses can vary depending on the specific genetic context. While our understanding of these interactions has progressed significantly, there are still aspects that require further exploration to understand the complexities of plant-ROS interactions.

### SA cross-talks with other plant hormones

**Fig. 4:**
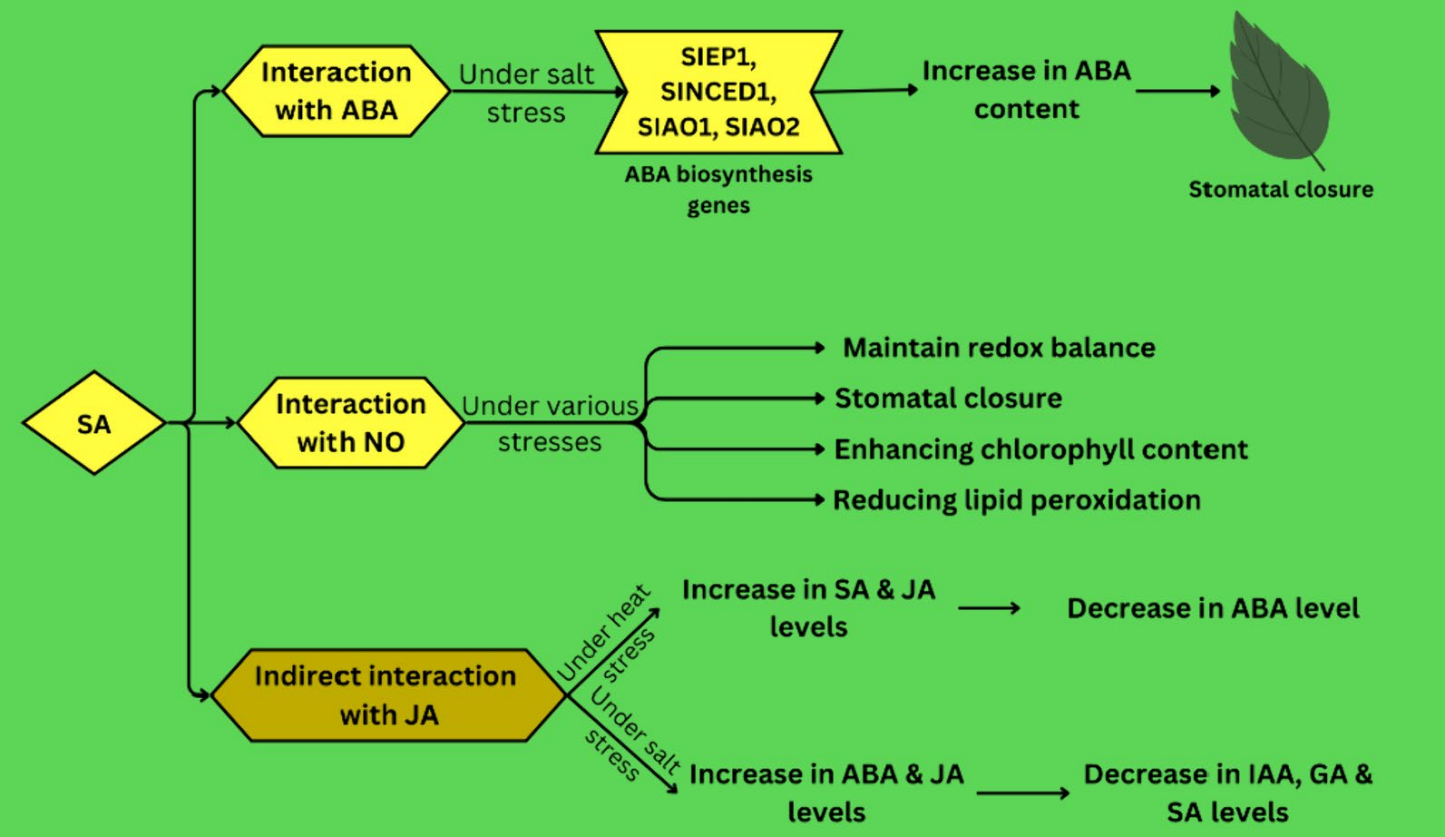
Cross-talks of SA with ABA, NO, and JA plant hormones under abiotic stress

In addition to ROS, other types of plant hormones play a role in the signaling pathway involving SA in plants. Various research studies have observed a connection between SA and ABA, SA and nitric oxide (NO), and SA with JA, especially during times of stress (Cao et al. 2011). All of these cross-talks aim to increase abiotic stress tolerance, Fig. 4.

The relationship between SA and ABA mostly is directly proportional and this cross-talk has a positive impact on tolerating abiotic stress. It was evident that when plants are treated with SA, which triggers an increase in ABA levels in plants such as in barley and tomato (Torun et al. 2020). This increase is mainly due to enhancing in expressing of ABA biosynthesis genes. Wang et al., (Wang et al. 2018b) reported elevated *NCED1* and *NCED2* gene expression in wheat leading to increased ABA content and reduced freezing stress. Szepesi et al. (Szepesi et al. 2009) reported increased in ABA biosynthesis genes (*SlZEP1, SlNCED1, SlAO1, and SlAO2)* expression after SA treatment in tomato under salt stress. However, when leaves of *A. thaliana* are exposed to ABA, it has an inhibitory effect on the transmission of SA signals through the SAR signaling pathway (Yasuda et al. 2008). It is suggested that the biosynthesis of ABA is a downstream event in the signaling process, influenced by the sensing of SA (Khan et al. 2019). Moreover, in response to drought stress, ABA facilitates cuticular wax biosynthesis through the transcription factor *MYB96*, which stimulates the production of SA as the super-active *myb96-1d* mutant shows increased expression of *sid2* the crucial enzyme in SA biosynthesis pathway. Also, the SUMO (small ubiquitin-like modifier) E3 Ligase 1 (*SIZ1*) negatively regulates the ABA-responsive gene *ABI3*, resulting in increased ABI3 protein levels in the *siz1* mutant (Miura et al. 2009). *SIZ1* also negatively modulates SA levels, leading to higher SA accumulation. (Corina Vlot et al. 2009; Miura et al. 2013). These effects can be reversed by introducing the *NahG* gene (Xu et al. 2015). More recently it was reported that SA and ABA signaling are integrating in stomatal guard cells under drought stressed condition (Prodhan et al. 2018; Li et al. 2023c); this integration is mediated by CBK pathway. The interaction between SA and ABA is still not fully understood, but it is clear that these two hormones play important roles in plant stress tolerance. Further research into this interaction is likely to lead to new strategies for improving plant resilience to stress.

Both SA and NO play crucial roles in maintaining redox balance in plants during responses to abiotic stresses (Rai et al. 2020). For instance, Applying SA and a NO-releasing compound significantly enhances the tolerance of certain plants to heat stress and nickel toxicity. Both NO and SA participates in the signaling pathway that leads to stomatal closure, with NO levels increasing through enzymes like those activated by SA (Wendehenne et al. 2004). Their combination effectively mitigates nickel toxicity in *Brassica napus* by facilitating proline accumulation, reducing lipid peroxidation, and enhancing chlorophyll content (Kamran et al. 2020; Ahmad et al. 2021). On the other hand, the NO-SA combination in *Arachis hypogaea* increases Cd concentration in the cell wall, thereby safeguarding organelles from its toxic effects (Shah et al. 2019). Moreover, the NO-SA interaction synergistically alleviates salt stress by enhancing divalent cations absorption (Verma et al. 2022).

The cross-talk between SA and JA is indirect and mostly it involves the interaction of other phytochromes. In a study involving heat-stressed soybean plants inoculated with *B. tequilensis*, the levels of endogenous JA and SA were significantly elevated, while the endogenous ABA level was markedly reduced (Aloo et al. 2023). Also, Salt stress on plants leads to an increase in the content of JA and ABA, but a decrease in the levels of other plant hormones like indole-3-acetic acid (IAA), gibberellic acid (GA), and SA in species like Iris hexagona and soybean (Wang et al. 2001).

### SA inducing mitogen-activated protein kinase

Mitogen-activated protein kinase (MAPK) is a specific type of protein kinase that targets threonine and serine amino acids. MAPKs play crucial roles in various cell functions and plant survival under a wide range of abiotic stress (Ji et al. 2017; Zhou et al. 2017). It was reported that MAPK cascade mediated are regulating H2O2 metabolism in tobacco and Arabidopsis (Xing et al. 2008).Research on *Arabidopsis thaliana* has indicated that MAPK cascade signaling is influenced by SA. Across various species, MAPK cascades have been implicated in signaling pathways triggered by abiotic stresses such as cold, salt, heat, UV, osmotic shock, heavy metals, and more (Raja et al. 2017). The components of MAPK cascades involved in different abiotic stresses.

SA-deficient mutants demonstrated a notable decrease, about 50%, compared with the wild-type expression level, in *AtMPK3* activity (Pál et al. 2013; Lin et al. 2021). SA activation led to the identification of a 48-kDa MAPK in tobacco, primarily phosphorylating myelin basic protein (MBP) (Lebrun-Garcia et al. 1998; Zhang and Klessig 1998). In turn, MAPKs influence SA levels in stressed plants. Remarkably, SA treatment boosted *TaMAPK4* transcripts in wheat during avirulent pathogen attacks, while downregulating the *TaMAPK4* gene led to decreased SA accumulation (Wang et al. 2018a). On the other hand, the StMKK1 protein negatively influenced SA-related signaling pathways during pathogen defense in potatoes. Although the *mpk4* mutant showed excessive SA accumulation, this wasn't solely responsible for its severe dwarf phenotype, as knocking down the *ICS1* gene failed to restore normal growth (Li et al. 2022b, 2023a). Furthermore, the connection between MPK4 accumulation and SA-regulated redox balance remains unclear and necessitates further investigation. Moreover, recently it was reported that SA triggers ROS signals in the stomatal guard cells which activate MPK genes-dependent pathway (MPK9 and MPK12) to regulate genes responsible for stomatal closure in Arabidopsis (Prodhan et al. 2018).

## Role of salicylic acid in mitigating abiotic stress in plants

### Effect of SA on plants under drought stress

Drought is one of the most important natural abiotic stresses that can negatively impact plant growth and development(Dai 2010). From an agricultural and physiological perspective, drought stress occurs when the available water in the soil is not enough to meet the needs of plants, which can eventually lead to a further decrease in soil moisture (Farooq et al. 2009b).

The exogenous application of SA at 100 mg/L significantly mitigated the negative effects of drought stress on *Brassica napus* plants (Ali et al. 2023). The application of SA reduced the effect of drought on leaf area chlorophyll, and gaseous exchange rate, (Table 2). It also reduced MDA contents, proline content, and sugar content, (indicators of lipid peroxidation and oxidative stress. The application of SA also minimized the deterioration ultrastructure caused by drought within the leaves (Ali et al. 2023). Feng et al. (Feng et al. 2023) reported that drought stress reduced the photosynthetic rate, stomatal conductance, and transpiration rate in flue-cured tobacco variety K326. However, SA spraying at 0.3 mM improved these parameters. SA also increased the activities of superoxide dismutase (SOD), peroxidase (POD), catalase (CAT) enzymes, and proline content, while decreasing MDA content (Table 2).

**Table 2:** Recent examples on the effects of SA on plants under various abiotic stressors

Host plant	Type of SA and treatment method	Stress symptoms on the plant without SA	Effect of SA on the plant	Reference
Drought				
Oilseed rape* (Brassica napus)*	Exogenous, foliar application (100 mg/L)	Reduction in leaf area, chlorophyll levels, gaseous exchange rate, mineral ions concentrations. Increase in MDA contents, Antioxidants levels, proline content, and sugar content in the leaves. Deterioration of ultra-structures within the leaves	Reduction in MDA contents, antioxidants, proline, sugar contents and minimization of the deterioration of ultra-structures	​​(Ali et al. 2023)
Flue-cured tobacco variety K326(*Nicotiana tobacum* L*.)*	Salicylic acid (SA) solution (0.3 mM) sprayed on the leaves every day for three days	Reduced the photosynthetic rate, stomatal conductance, and transpiration rate. Enhanced the SOD, POD, CAT, MDA, proline and protein content	Improved the photosynthetic rate, stomatal conductance, and transpiration rate. Increased the activities of SOD, POD, CAT, protein, and proline content while the MDA content decreased by 23.89%. Increased drought tolerance of tobacco by modulating the expression level of the genes associated with photosynthesis, carbon metabolism, and photosynthesis-antenna proteins	(Feng et al. 2023)
white mulberry (*Morus alba L*)	Exogenous, foliar application (0.5 and 1.0 mM)	Decreased plant growth, leaf, stems, and roots dry biomass. Decreased chlorophyll a, b, and carotenoid contents, leaf gas exchange parameters. Increased electrolyte leakage, MDA contents, hydrogen peroxide, and superoxide radicals	Increased leaf, stems, and roots dry biomass. Increased chlorophyll a, b, carotenoid contents, leaf gas exchange parameters, Increased proline content, total soluble sugar, total phenolic content, soluble protein and SOD, POD, CAT, and ascorbate peroxidase. Decreased electrolyte leakage, MDA contents, hydrogen peroxide, and superoxide radicals	(Zafar et al. 2023)
Grape tomato (*Solanum lycopersicum*)	Exogenous, foliar application (Several concentration)	Reduced plant height, leaf area, and yield	Increased fruit yield, height and leaf area	(Chakma et al. 2021)
Maize (*Zea mays*)	Exogenous, pre-harvest foliar spraying (1.0 μM)	Membrane lipid peroxidation and wilting of leaves	Reduced membrane lipid peroxidation, increased plant height, and dry mass, less wilting of leaves	​​​ (Saruhan et al. 2011)
Wheat (*Triticum aestivum*)	Exogenous, foliar application (0.5 mM)	Membrane lipid peroxidation, leaf wilting	Reduced membrane lipid peroxidation, increased plant height and dry mass, reduced leaf wilting	(Kang et al. 2012)
Rice (*Oryza sativa*)	Exogenous, priming (soaking seedlings) (50–100-150 mg/L)	Reduced seedling fresh and dry weights, and decreased photosynthesis	Increased proline and GB content, increased fresh and dry weights, increased antioxidant activity	​​(Farooq et al. 2009a)
Heat				
Oilseed rape (*Brassica napus*)	Exogenous, foliar application (50 ppm)	Heat stress-induced lipid membrane damage, reduced leaf chlorophyll contents, net photosynthetic rate, and lint yield	SA treatment increased leaf SOD, CAT activity, chlorophyll contents, net photosynthetic rate, number of sympodial branches, boll weight and fiber quality components	(Sarwar et al. 2018)
Tomato (*Solanum lycopersicum)*	Exogenous, pre-harvest foliar application (1 mM)	Reduced chlorophyll content, reduced leaf water potential, oxidative damage	Increased antioxidant enzyme activity, reduced MDA and H_2_O_2_, proline accumulation	​​(Shah Jahan et al. 2019)
Alfalfa (*Medicago sativa*)	Exogenous, foliar application (0.25–0.5 mM)	Leaf chlorosis, reduced height, increased MDA content	Improved plant morphology, growth and membrane stability	​​(Wassie et al. 2020)
Rice (*Oryza sativa*)	Exogenous, foliar application (100 mg/L)	Decreased root and shoot length, decreased fresh and dry weights, decreased protein and sugar content	Improved growth, increased fresh and dry weights, protein content, and inorganic minerals content	​​(Akasha A et al. 2019)
Cucumber (*Cucumis sativus*)	Exogenous, foliar spraying (1 mM)	Lower electrolyte leakage parameter, lower H_2_O_2_ levels, lower catalase activity and lipid peroxide levels, and lower Fv/Fm chlorophyll a fluorescence value	Induced heat tolerance, higher electrolyte leakage parameter, higher H_2_O_2_ levels, higher catalase activity and lipid peroxide levels, and higher Fv/Fm chlorophyll a fluorescence value	​​(Shi et al. 2006)
Chilling				
Hami melons (*Cucumis melo)*	Exogenous, dipping post-harvest (1–3-9 mM for 5 min)	Chilling injury, faster browning, reduced weight	Firmer melons, delayed browning, more storage time	(Song et al. 2022)
Peach (*Prunus persica)*	Exogenous, dipping post-harvest (1 μmol/L for 15 min)	Faster browning, faster softening	Delayed browning, increased expression of genes related to cold tolerance	(Zhao et al. 2021b)
Grapes (*Vitis vinifera)*	Exogenous, pre-harvest spraying (1–2-3–4 μmol/L at two stages)	Increased rate of rot and decay, shorter shelf life	Higher-quality berries, prolonged shelf life	(Gomes et al. 2021)
Kiwi (*Actinida deliciosa*)	Exogenous, post-harvest soaking (1 mM for 5 min)	Severe chilling injury, deep flesh color, water stains,	Enhanced phenolic accumulation, increased PAL activity via up-regulation of 2 genes encoding PAL, alleviated chilling injury symptoms	(Niu et al. 2023)
Tomato (*Solanum lycopersicum*)	Exogenous, pre-harvest spraying (1–2-4 mM three consecutive weeks before harvest)	Faster weight loss, rapid decrease of ascorbate peroxidase activity, quicker loss of firmness	Better maintenance of fruit firmness, higher lightness	(Baninaiem and Dastjerdi 2023)
Salinity				
Damask rose (*Rosa damascena*)	Exogenous, foliar application (0.5–1-2 mM)	Shortened root length, decreased leaf area, reduced chlorophyll content, increased MDA content	Neutralization of salt stress, increased root depth, larger leaf area, normal chlorophyll levels, increased POD and APX activity, and antioxidant gene expression	(Omidi et al. 2022)
Saffron (*Crocus sativus*)	Exogenous, foliar application (1.5 mM)	Decreased root length, decreased corm number, decreased flower number	Mitigation of salt stress, oxidative damage, and osmotic stress and increased flower number	(Feizi et al. 2021)
Pepper (*Capsicum annuum* L.)	Exogenous, foliar application (0.5 mM)	Reduced plant growth, oxidative stress, decreased chlorophyll content	Improved tolerance to salinity, enhanced plant growth, phenotypic appearance, reversed effects of oxidative stress, improved chlorophyll content	(Kaya et al. 2020)
False wheatgrass (*Leymus chinensis*)	Exogenous, priming (soaking seedlings) (0.1 mmolL^−1^—0.5 mmol/L)	Lower germination rate, plasma membrane damage, ion toxicity, and damage	Increased antioxidant enzyme activity, enhanced germination and viability, mitigated damage to the plasma membrane, ROS modulation	(Hongna et al. 2021)
Tomato (*Solanum lycopersicum*)	Exogenous, root application (100 mg/L)	Reduced leaf area and height, low shoot weight	Increased leaf area, plant height, shoot weight and proline concentration	(Souri and Tohidloo 2019)

In *Arabidopsis thaliana*, SA-accumulating mutants (*cpr5* and *acd6)* exhibited stomatal closure and improved drought tolerance. This was attributed to SA-mediated induced expression of PR genes, such as *PR1*, *PR2*, and *PR5* ​(Liu et al. 2013). In wheat, 78 proteins were identified that were potentially involved in SA signaling under drought stress. These proteins were involved in major physiological functions, such as photosynthesis, carbohydrate metabolism, protein metabolism, stress and defense, energy production, signal transduction, and toxin metabolism (Kang et al. 2012).

### Effect of SA under temperature stress

One of the most detrimental stressors on plants and a major side effect of global warming is extreme temperatures. Whether high temperature (HT) or chilling, both extremes are sure to stunt the growth and development of the plant.

### Role of SA in heat stress tolerance

**Fig. 5:**
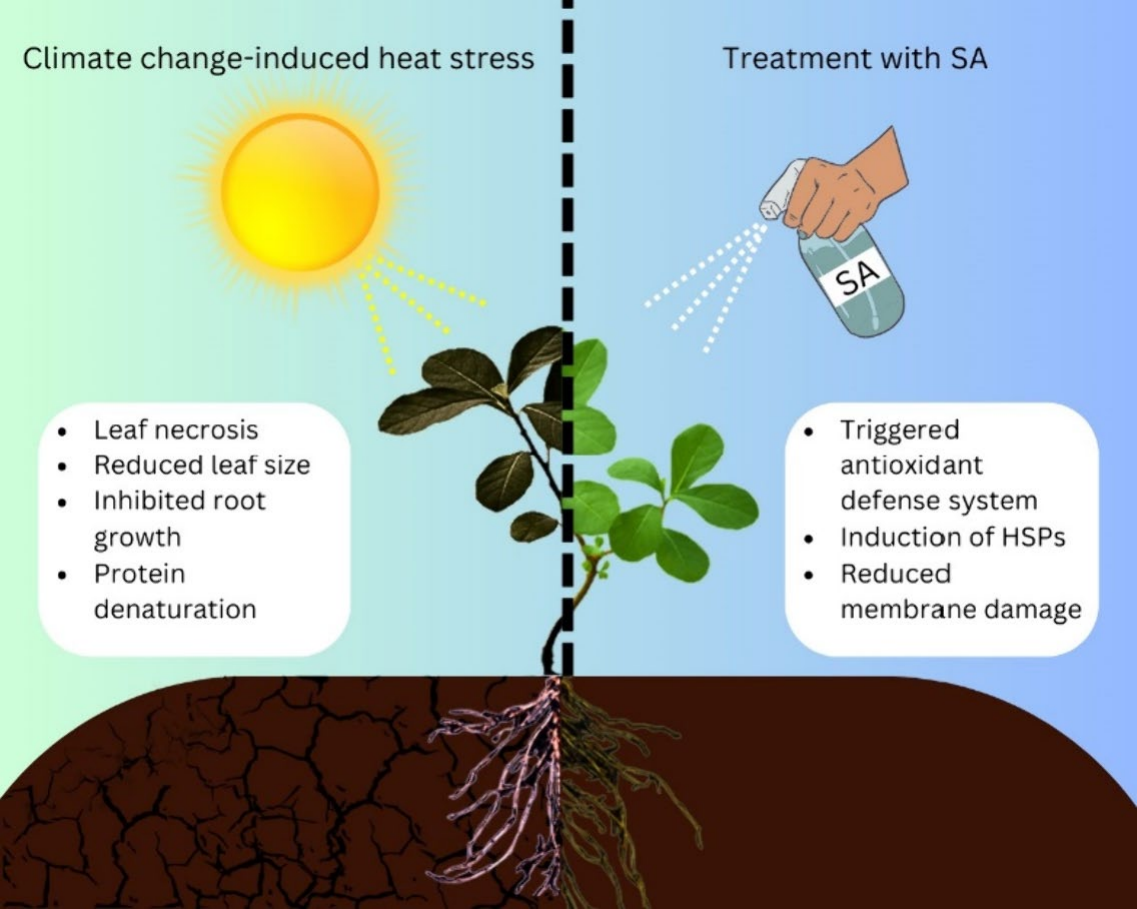
Effect of climate change-induced stress on plants (left) and effect of SA treatment on heat stress-exposed plants (right)

Heat stress (HT) is a significant environmental stress that can hinder plant growth, metabolism, and productivity (Zhao et al. 2020). Global temperatures increasing by 1.5 degrees Celsius by 2050 and 2 to 4 degrees Celsius by 2100 accordingly HT is a growing concern for crop production (Zhao et al. 2020). HT can induce physiological, biochemical, and molecular changes, disrupting membrane integrity and protein denaturation (Fig. 5) (Levitt 1980).

SA is thought to induce heat tolerance in plants by activating many genes involved in stress response, including genes encoding antioxidant enzymes and heat shock proteins (Suzuki et al. 2008). *MBF1c*, a highly conserved transcriptional coactivator, is upregulated by heat stress (Nazar et al. 2017). ​Suzuki et al. (Suzuki et al. 2011)​ reported that *MBF1c* is a key regulator of basal thermotolerance and provided evidence for the existence of a coordinated heat stress response network involving SA signaling pathways under the control of *MBF1c*. *MBF1c* is induced by heat stress and binds to the promoter of a gene encoding SA. Furthermore, in Arabidopsis *ATMBF1c* recognizes and regulate stress-responsive gene dehydration-responsive element (*DRE*)-binding protein 2A (*DREB2A*). It is evident that expression of *PaMBF1c* of *Antarctic Moss*) enhanced heat stress tolerance in Arabidopsis (Alavilli et al. 2017).

SA is thought to help plants tolerate HT stress by activating genes involved in stress tolerance. (Khan et al. 2013)​ found that SA treatment under heat stress increased the production of proline, which increased the osmotic potential of the plants. This enabled the plants to uptake more water, which had a positive influence on stomatal aperture and photosynthetic machinery. In cucumber plants, foliar spraying with 1 mM SA induced heat tolerance, as evidenced by the lower electrolyte leakage parameter, lower H_2_O_2_ levels, higher catalase activity, and lipid peroxide levels, and higher Fv/Fm chlorophyll a fluorescence value ​(Shi et al. 2006). SA has been found to increase the photosynthetic rate in grape leaves under heat stress ​(Wang et al. 2010)​. It can also alleviate heat-induced damage in plants by upregulating the antioxidant system ​(Fig. 5) (Wang and Li 2006).

Several investigations had been conducted with the aim of deciphering the effect of SA on heat tolerance from the molecular and biochemical point of view. SA stabilizes the trimerization of heat shock transcription factors (HSFs) and helps them bind to the heat shock element (HSE) in the promoter of heat shock protein (HSP) genes (Andrási et al. 2021; Haider et al. 2022; Wang et al. 2010)​ found that SA did not affect the level of heat shock protein 21 (*HSP21*) in grape leaves before heat stress. However, the HSP21 defense signal increased in both SA-treated and control leaves during heat stress. (Wang and Li 2006)​ found that spraying a 0.1 mM solution of SA on young grape leaves increased heat tolerance by reducing the levels of thiobarbituric acid reactive substances (TBARS) and relative electrolyte leakage and increased the activity of antioxidant enzymes. These results suggest MBF1c, a highly conserved transcriptional coactivatthat SA can induce intrinsic heat tolerance in plants. ​(Pan et al. 2006) found that blocking SA synthesis in plants under heat stress decreased both the endogenous SA content and the level of heat tolerance.

Liu et al. (Liu et al. 2008) found that SA can induce heat tolerance in plants by regulating the activities of plasma membrane H^+^-ATPase and Ca^2+^-ATPase. Furthermore, SA-induced thermotolerance is associated with the Ca^2+^-calmodulin (CaM) system in grape plants. SA increased the activity of H^+^-ATPase in grape leaves, which helped to maintain the stability of the plasma membrane and protect cells from heat damage. The increase in the Ca^2+^ level in the cellswas the reason behind Ca^2+^-CaM system activation ​(LIU and HUANG 2005). This further clarifies the role of SA in stimulating the activity of Ca^2+^-ATPase, which helped to regulate the Ca^2+^ level in the cytoplasm and prevent heat injury.

The role of the genetics underlying SA biosynthesis and functions in plants facing heat stress is still under study. However, According to (Nazar et al. 2017) recent studies, SA plays an important role in heat stress tolerance by activating genes that encode proteins that help the plant cope with heat stress, such as HSPs and antioxidant enzymes. The genes that encode SA are also involved in the regulation of heat stress response, and their overexpression can improve the heat tolerance of plants. More research is needed to fully understand the role of the genetics of salicylic acid in heat stress tolerance, but these studies suggest that it is a promising target for the development of new crop varieties that are more tolerant to heat stress.

### Role of SA in chilling stress tolerance

Extremely low temperatures could result in chilling injury to the plant. A chilling temperature denotes a temperature as low as possible without freezing (Kang and Saltveit 2002). Plants exposed to these low temperatures are at risk of suffering from slower seed germound that SA can induce heat tolerance in plants by regulating the aination and growth, a slower rate of photosynthesis, as well as stiffening and deterioration of the plasma membrane (Saleem et al. 2020). Cold stress also decreases water and nutrient uptake, hence putting the plant at risk of starvation (Saleem et al. 2020). The low temperatures lead to decreased plasma membrane fluidity due to disruption of the ratio and composition of plasma membrane lipids and proteins (Saleem et al. 2020). This causes the membrane to go from fluid-like to gel-like. The most visible symptom of chilling injury manifests itself as internal browning and softening of the plant (Zhao et al. 2021b).

Exogenous SA has been proven in numerous investigations to be effective in mitigating cold stress and maintaining fruit quality (weight loss, discoloration, softening, etc.). This might be because SA can promote protein synthesis and stimulate enzyme activity, which could wake the plant from its dormant state (Liu et al. 2022). SA also can enhance water and nutrient uptake in plants, although the mechanism under chilling stress is still uncovered (Saleem et al. 2020). Furthermore, SA has been shown to delay the accumulation of ethylene (ET) in fruit, which delay fruit senescence, a common symptom of chilling injury in plants (Chen et al. 2023). (Song et al. 2022) showed that soaking post-harvest Hami melons in SA improved their cold tolerance and extended their storage time, (Zhao et al. 2021b) reported that SA treatment delayed the internal browning of the peaches. Overall, evidence of the ability of SA to mitigate cold stress, whether it was applied pre-harvest or post-harvest has been documented heavily.  (Zhao et al. 2021b) reported that SA treatment delayed the internal browning of the peaches and mitigated the overall effects of chilling injury by increasing the total soluble sugars via increasing the synthesis of sucrose and the expression of genes related to sucrose synthesis, such as *Neutral Invertase 2* (NINV2), *Sucrose Phosphate Synthase 4* (*SPS4*), *Sucrose Synthase 2* (*SuSy2*), and *Sucrose Transporter 1* (*SUT1*). SA treatment also increased the expression of genes involved in cold-stress response, *DREB1A* and *DREB2A* (Zhao et al. 2021b). Exogenous SA treatment was also involved in the upregulation of genes involved in the cold signaling pathway, such as *C-repeat Binding Factor 1* (*CBF1*), and *Inducer of CBF expression 1* (*ICE1*) in cucumber plants (Fu et al., 2021). Overall, evidence of the ability of SA to mitigate cold stress, whether it was applied pre-harvest or post-harvest has been documented heavily. Table 2 displays recent examples of SA treatment and its effects on plants under chilling stress.

### Effect of SA on plants under salinity stress

Salinity is regarded as one of the most detrimental abiotic stressors to plants and crops. Salinity denotes an elevated concentration of soluble salts present in the soil (Singh and Gautam 2013). Plants exposed to salinity are threatened with various deleterious effects such as osmotic stress, reduced chlorophyll amounts, altered metabolic activity, decreased size, and shortened height of the plant (Wani et al. 2017; Liu et al. 2022). Salinity has also been linked to the formation of ROS, leading to oxidative stress and eventually DNA damage, as well as lipid peroxidation and enzyme inactivation (Hayat et al. 2010; Wani et al. 2017). Salinity has also been shown to decrease endogenous SA content in plants, e.g., tomatoes (Molina et al. 2002; Liu et al. 2022).

SA mainly support plants to tolerate salinity tolerance by modulating key salinity stress responsive genes. SA was found to increase the transcript of *1-aminocyclo propnae 1-carboxylic acid synthase* (*ACS),* salt overly sensitive 1 (sos1), sodium-hydrogen exchanger (NHX1), and high-affinity K^+^ transporter (HKT1;2) under salinity stressed tomato plants. Furthermore, those SA treated plants exhibited reduced electrolyte leakage and sodium ion (Na^+^) content and increased endogenous proline and potassium ion (K^+^) content, and K^+^/Na^+^ ratio (Szepesi et al. 2009). In addition, SA prevented K^+^ loss via guard cell outward-rectifying K^+^ (GORK) channels. This reduction is NPR1-dependent as npr1-5 mutant is unable to control K^+^ leak (Jayakannan et al. 2013). Moreover H^+^ ATPase activity was increased in SA pretreated Arabidopsis plants subsequently there were a reduction in NaCL-induced K^+^ efflux through KOR channels resulting in tolerance to salinity stress (Jayakannan et al. 2013).

As a vital regulator of sodium influx and efflux, SA helps alleviate salt stress (Liu et al. 2022). Omidi et al., (Omidi et al. 2022) demonstrated that foliar application of SA in concentrations as little as 0.5 mM was still able to neutralize the salt stress and increase the expression of antioxidant genes in the damask rose. When (Hongna et al. 2021) soaked the seeds of false wheatgrass (*Leymus chinensis*) in SA solution and then subjected them to salt stress, the seeds exhibited improved germination and viability, as well as alleviated plasma membrane damage and ROS modulation. Although not as efficient as foliar pretreatment, treating tomato roots using SA still resulted in alleviated salt stress (Souri and Tohidloo 2019). Table (2) displays a summary of recent investigations reporting the effect of SA on various plants that were subjected to salt stress.

SA was also found to increase the amount of osmolytes (proline) present in plants, which acts on in mitigating salt stress as osmolyte accumulation represents a vital adaptive response to stress in plants (Hayat et al. 2010; Singh and Gautam 2013). Proline represents a very important stress amino acid that accumulates under salt-stressed conditions. This is because it plays a vital role in regulating osmotic balance, which could help in alleviating salt stress (Kang and Saltveit 2002). Furthermore, exogenous application of SA was found to maintain membrane stability and integrity as well as increase the rate of photosynthesis (Hayat et al. 2010; Singh and Gautam 2013).

### Effect of SA on plant response to other abiotic stresses

Sunlight, essential for plant growth, can be harmful to plants, particularly UV light, which can damage DNA and proteins​ (Müller-Xing et al. 2014). Climate change's frequent heatwaves have increased UV radiation levels (Bernhard et al. 2020)​. UV radiation is divided into UVA, UVB, and UVC regions, with UVC being the most harmful. SA can modulate antioxidant levels, detoxify superoxide radicals, prevent oxidative damage, increase photosynthetic rate, pollen viability, leaf phenolic concentration, and yield in UV-B-stressed plants ​(Karioti et al. 2008; Mohammed and Tarpley 2013; Li et al. 2014). The SA-mediated modulation of UV-induced oxidative stress was thought to be largely influenced by the activation of antioxidant enzymes in Capsicum annuum leaves ​((Khan et al. 2015))​. Increased photosynthetic rate, pollen viability, leaf phenolic concentration, and yield have also been reported in UV-B-stressed rice plants (Mohammed and Tarpley 2013; Khan et al. 2015). SA has been shown to decrease the level of chromosome aberrations caused by UV-B radiation in meristematic root tip cells ​(Rančelienė and Vyšniauskienė 2012; Khan et al. 2015). Exogenously applied SA has been reported to significantly improve photosynthetic function and its related variables in plants exposed to UV-B radiation ​((Karioti et al. 2008; Khan et al. 2015). UV-C radiation has also been shown to upregulate the transcription of the SA induction deficient 2 gene, which codes for the SA biosynthetic ICS1 enzyme (Martínez et al. 2003; Khan et al. 2015). Additionally, the SA-dependent pathway has been reported to control the upregulation of the PR proteins (*PR-1*, *PR-2*, and *PR-5*) in UV-B-exposed transgenic NahG A. thaliana plants (Surplus et al. 1998; Khan et al. 2015).

Ozone, a strong oxidizing agent, penetrates mesophyll cells in plants through the leaf stomata; this leads to the generation of ROS after reacting directly with proteins and lipids, a process that could take mere minutes (Hara et al. 2012; Hasan et al. 2021; Leisner et al. 2022). The ozone-induced ROS then leads to the formation of an oxidative burst in the apoplast, wherein the plant undergoes lipid peroxidation and apoptosis, leading to leaf necrosis (Hara et al. 2012; Leisner et al. 2022; Ramya et al. 2023). The result of the necrosis disrupts the rate of photosynthesis, reproductive capacity, stomatal conductance, and the amount of chlorophyll in plants (Hasan et al. 2021; Ramya et al. 2023). Thereby affecting the growth rate, yield, and quality of crops (Ramya et al. 2023). Additionally, ozone-exposed plants are more vulnerable to biotic stressors (Leisner et al. 2022).

Ozone exposure has been shown to cause SA accumulation in plants (Horváth et al. 2007b; Hara et al. 2012). SA does not work alone in defending against ozone stress; instead, it acts both synergistically and antagonistically with other phytohormones belonging to the same signaling network including, ET and JA (Hara et al. 2012; Hasan et al. 2021; Leisner et al. 2022). Although ET responds faster relative to SA and JA (Grulke and Heath 2019). ET, much like SA, has been shown to defend plants against both biotic and abiotic stressors, although their role in mitigating ozone stress is not entirely understood. ET has been shown to increase SA accumulation via mediated regulation of the *PAL* gene (Khan et al. 2015). Whereas SA-treated plants showed increased ET production (Hara et al. 2012). On the other hand, JA impeded the oxidative burst caused by ozone upstream of SA accumulation (Leisner et al. 2022).

## Genome editing and advanced genetic engineering approaches for understanding the role of SA and abiotic resistance

To explore underlying mechanism of SA to regulate climate change induced abiotic stresses, more investigations at physiological and molecular levels are required. Genetic engineering considers a great tool in revealing and filling the gaps in plant SA biochemical pathways. Genome editing holds a great opportunity to decipher and enhance our knowledge regarding the genetics involved in the role of SA. Genome editing is a technology that can accurately pinpoint a specific genomic sequence and alter that sequence for genetic improvement of biological studies (Zhang et al. 2021a). Mainly to knock out or in the function of a gene or a sequence (Abdelrahman and Zhao 2020; Tang et al. 2021; Wei et al. 2021; Abdelrahman et al. 2021b). Many genome editing techniques have been used on crops to enhance their stress tolerance and quality. Among others, clustered regularly interspaced short palindromic repeats and CRISPR-associated protein 9 (CRISPR/Cas9), has proven to be the most versatile and highly effective (Abdelrahman and Zhao 2020; Abdelrahman et al. 2021b). Also, transformed plants with foreign genes play an important role in understanding gene function either through overexpression or being inserted as a sole copy in a test genetic background.

The *NPR1* gene is the SA receptor. Its role in plant immunity to pathogens has been well described (Wu et al. 2012). Yet, its role in regulating the plant response to abiotic stress is poorly understood. To decipher its role under drought, CRISPR/Cas9 was utilized to knock out the *NPR1* gene in tomatoes. Mutant plants exhibited sensitivity to drought stress and lower levels of drought-related gene transcripts, including *SlGST*, *SlDHN*, and *SlDREB* (Li et al. 2019). *SlNPR1* knocked out mutants were sensitive to drought stress with increased H_2_O_2_ and MDA content, developed oxidative damage and suppressed antioxidant genes.

Furthermore, the NPR1-dependent SA signaling pathway was proved to be crucial for alleviating salt and oxidative stresses in *A. thaliana* (Jayakannan et al. 2015). Expression of *AtNPR1* in transgenic tobacco plants enhanced the tolerance to oxidative stress. The tolerance was associated with a constitutive upregulation in *PR1*, *PR2*, *PR5*, *APX,* and *SOD* genes (Srinivasan et al. 2009). In contrast, its heterologous expression, *AtNPR1*, in rice plants exhibited high sensitivity to salt and drought stresses accompanied by negative regulation salt and drought stress responsive genes; (*rab21*, *salT,* and *dip1*) (Quilis et al. 2008). Further, molecular and physiological studies are required to explain these contradictory results questioning the role of the *NPR1* gene. While overexpression of the *PR1* gene enhanced the tolerance of the transgene tobacco plants to heavy metals (Sarowar et al. 2005). Another study concluded that transgenic plants where the *AhSIPR10* gene was overexpressed showed tolerance to heavy metal, salt, and drought stress (Jain et al. 2012).

Furthermore, in tomatoes the expression rates of SA-related genes, *SlPR1* and *SlPR2,* were increased in *SlHyPRP1* edited plants. These edited plants showed multiple abiotic stress tolerance (Tran et al. 2023). Disrupting the *OsCYP71A1* blocked serotonin biosynthesis and greatly increased salicylic acid levels in knocked-out rice plants.

CRISPR-Cas9 technology has facilitated the enhancement of plant stress tolerance through targeted gene modifications. In the context of drought stress, genes associated with stress response have been edited in various crops, such as the knockout of ABA signaling-related genes like *OST2, miR169a, AVP1, AREB1, and TRE1* in Arabidopsis (Table 3) (Osakabe et al. 2014; Park et al. 2017; Roca Paixão et al. 2019), and genes involved in drought-responsive pathways like *DERF1, PMS3, MSH1, MYB5, SPP, SRL1, SRL2,* and *ERA1* in rice (Shi et al. 2017; Liao et al. 2019; Ogata et al. 2020; Usman et al. 2020).

**Table 3:** Recent instances for the use of CRISPR/Cas9 technology in investigating key genes associated with drought stress tolerance

Gene name	Crop	Reference
*AREB1*	Arabidopsis *(Arabidopsis thaliana)*	(Roca Paixão et al. 2019)
*mir169a*	Arabidopsis *(Arabidopsis thaliana)*	(Zhao et al. 2016)
*GT79B2, UGT79B3*	Arabidopsis *(Arabidopsis thaliana)*	(Li et al. 2017)
*PtoMYB216*	Arabidopsis, Poplar	(Xu et al. 2017a)
*ARGOS8*	Maize *(Zea mays)*	(Shi et al. 2017)
*OsSAPK2*	Rice *(Oryza sativa)*	(Lou et al. 2017)
*SlNPR1*	Tomato *(Solanum lycopersicum)*	(Li et al. 2019)
*slmapk3*	Tomato *(Solanum lycopersicum)*	(Wang et al. 2017)

CRISPR/Cas9 based genome editing has been utilized successfully in different research investigations for several abiotic stress responsive genes. The heat stress response has also been augmented through CRISPR-Cas9-mediated gene editing. Heat-inducible rice mutants have been created, and heat stress sensitivity has been increased in tomatoes by editing genes such as *MAPK3* and *agamous-like 6* (Nandy et al. 2019; Bouzroud et al. 2020). Overexpression of the *HSPS* gene, which increases osmolyte levels and prevents cell protein damage, can also be used to make plants more resistant to heat (Debbarma et al. 2019). The protein kinase SAPK6 and the transcription factor OsbZIP46CA1 in rice have also been shown to increase the capacity for responding to heat stress (Chang et al. 2017). Cold stress mitigation has been achieved through CRISPR-mediated knockout of genes like *OsPRP1* and *OsMYB30* in rice, as well as modulation of the *CBF* genes in Arabidopsis and tomato (Li et al. 2022c).

Salinity stress tolerance has been improved by targeting genes like *OsRR22* and *OsPQT3* in rice, involved in cytokinin metabolism and salt stress response respectively (Table 4) (Takagi et al. 2015; Zhang et al. 2019; Alfatih et al. 2020). Furthermore, CRISPR-Cas9 technology has been employed to address heavy metal stress. Genes associated with heavy metal transport and detoxification, including *oxp1* in Arabidopsis and *OsNRAMP1* in rice, have been edited to enhance plant resistance to heavy metals like Cd and lead (Wang et al. 2017; Hasanuzzaman et al. 2019; Chu et al. 2022).

**Table 4:** CRISPR-mediated genome editing for uncovering genes involved in salinity stress responses

Species	Target Gene	Improved Trait	Reference
Wheat *(Triticum aestivum)*	*Two HAG homologs*	Enhanced salinity tolerance more chlorotic leaves	(Zheng et al. 2021)
Rice *(Oryza sativa)*	*GTg-2*	Reduced sensitivity of salt	(Liu et al. 2020a)
Arabidopsis *(Arabidopsis thaliana)*	*SAUR41*	Enhanced salinity tolerance more chlorotic leaves	(Qiu et al. 2020)
Rice *(Oryza sativa)*	*RR22*	Enhance salinity tolerance	(Zhang et al. 2019)
Rice *(Oryza sativa)*	*PQT3*	Enhance salinity tolerance	(Alfatih et al. 2020)
Rice *(Oryza sativa)*	*PIL14*	Reduced sensitivity of salt	(Mo et al. 2020)
Arabidopsis *(Arabidopsis thaliana)*	*C/VIF1*	Enhanced salinity tolerance more chlorotic leaves	(Yang et al. 2020)

While there have been a handful of studies to genetically amplify the amount of SA in the plant to induce resistance against pathogens (Ma et al. 2018; Tezuka et al. 2021; Liu et al. 2023), little or no studies have been published to explore the possibility of inducing resistance against abiotic stressors using novel genetic techniques. This leaves the field open to exploration and potential new findings that may breed new plants with an enhanced tolerance for abiotic stressors.

## Conclusion and future perspectives

This comprehensive review provides information about the genetic and physiological basis of SA's in supporting plants to withstand abiotic stress in plants. The review highlights SA's significance as a versatile safeguard for plants against various abiotic stressors. It has the potential to enhance plant resilience in the face of challenging environmental and climatic stress conditions. By exploring the complexities of SA production pathways, this review sheds light on the promising mechanisms underlying the SA enhanced plant resistance to climate change related abiotic stresses, such as cross-talks with other phytohormones, orchestrating plant responses to stress, and employing multiple mechanisms to enhance stress tolerance. It is evident that SA pathway is sensitive to the alteration of climatic factors. SA biosynthetic pathways mediated by both isochorismate synthase (ICS) and phenylalanine ammonia lyase (PAL) are regulated by abiotic derived from changes in the climate. The SA transcriptional coactivator NPR1 is positively regulated by chilling condition and suppressed by drought and high humidity(Rossi et al. 2023). The changing of abiotic stress impacts the key component of SA pathway through its biosynthesis levels, signaling, metabolism and transport in plants.

**Fig. 6:**
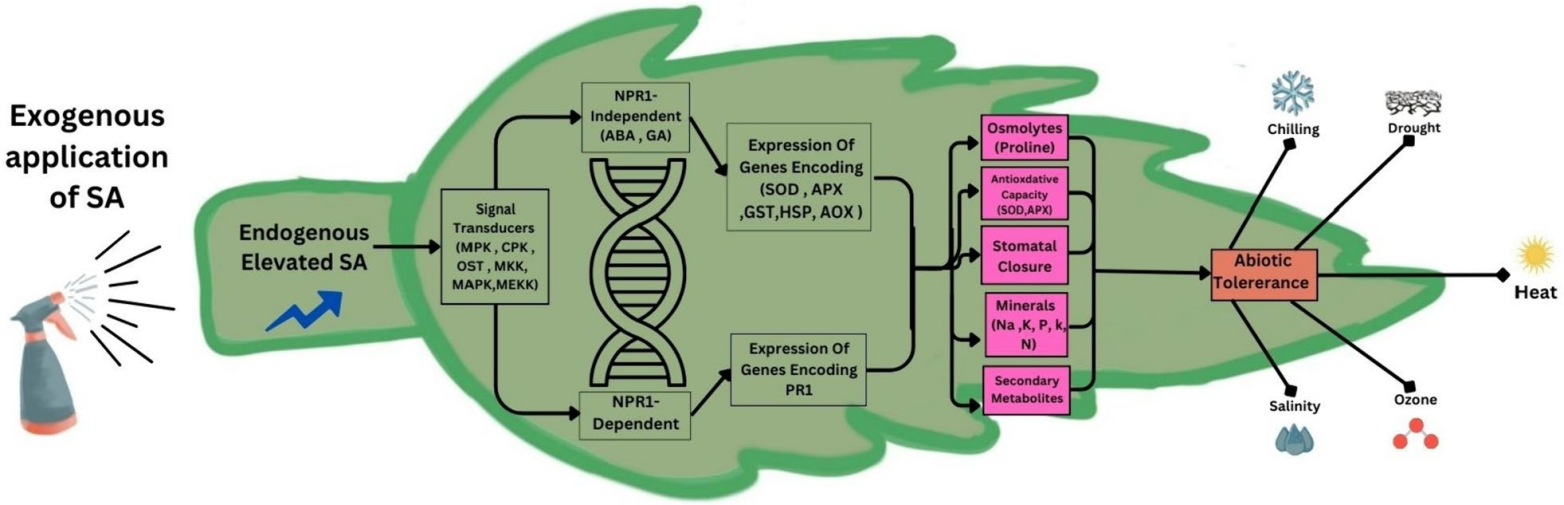
SA induction of various mechanisms to induce tolerance to abiotic stress

While significant knowledge has been obtained on the biosynthesis of SA and its role in stress tolerance, as it is only a recent discovery, there are still significant knowledge gaps that necessitate further investigation. For example, there is a hypothesis suggesting the involvement of a BA2H enzyme in converting BA into SA, but the precise identification of the responsible gene remains elusive. Over the years, multiple genetic mechanisms responsible for the role of SA in mitigating stress have been uncovered. Despite what we know, many mechanisms have yet to be fully understood and uncovered. For instance, how does exogenous SA interact with endogenous SA? What is the exact mechanism by which SA interacts with other phytohormones? Those and many other questions require further research to unravel the precise mechanisms underlying SA-mediated climate change mitigation and explore its potential applications in agriculture and ecosystem management.SA has been shown to alleviate the adverse effects caused by various climate change-related abiotic stressors. Exogenous SA treatment was found to induce tolerance to abiotic stressors via the induction of various signal transducers, leading to the activation of the broad-spectrum stress response, SAR, ultimately leading to the generation of signaling responses to stress (Fig. 6). While the physiological role of SA in mitigating abiotic stress has been documented broadly, few have explored the proteomic, transcriptomic, and genomic sides. Perhaps utilizing novel bioinformatics tools could help reveal the complexity of the SA signaling network and its defensive role.

Further future investigations should be employed by combining the different fields of research. Transcriptomics analysis of different genotypes under different stress conditions compared to normal conditions together with exogenous application of SA should be employed. Furthermore, comparative analysis of SA pathway involved genes in different plant species should be conducted to decipher the differences of the SA effect under different stresses in those species. This should be based on the available bioinformatic tools and online databases. The output of these research investigations should be examined by the new biotechnological tools of genome editing to study the effect of knocking in/out different genes on the genetic response of different SA linked genes. There is a great potential to use novel genetic approaches, such as genome editing, to reveal new discoveries in the genetic pathway of SA. Genome editing has been utilized successfully to breed plants for diverse effects (Abdelrahman et al. 2021a and Abdelrahman et al. 2021b). Genome editing via CRISPR/Cas9 provides a versatile tools and mechanisms to develop plants better equipped to withstand climate challenges and reduce the threat of food insecurity also remains unexplored.

## Data Availability

Not applicable.

## Abbreviations

SA: Salicylic acid
CKs: Cytokinin
ABA: Abscisic acid
ET: Ethylene
JA: Jasmonates
Pro: Proline
GB: Glycine betaine.
PAL: Phenylalanine ammonia-lyase pathway
ICS: Isochorismate pathway
ICS1: Isochorismate synthase 1 gene
tCA: Trans-cinnamic acid
BA: Benzoic acid
NPR: *Nonexpresser of pathogenesis-related* Genes
PEP: Phosphoenolpyruvate
E4P: Erythrose-4-phosphate
EDS 5: *enhanced disease resistance5* Gene
PBS3: *avrPPHB susceptible 3* Gene
GH3: Glycoside hydrolase 3
EPS: *enhanced pseudomonas susceptibility* Genes
SGE: SA-β-D-Glucose Ester
SAG: SA-2-O-β-D-glucoside
SAR: Systemic acquired resistance
MeSA: Methyl salicylate
*AtAIM1*: Abnormal inflorescence meristem 1
Ba2H: BA 2-hydroxylase enzyme
SARD: *Systemic Acquired Resistance Deficient*
TCP: Teosinte branched1/cycloidea/proliferating cell factor.
CBP: CaM-Binding Protein.
EIN: *Ethylene Insensitive*
CPR: *constitutive expresser of pathogenesis-related* Genes
ACD: *accelerated cell death gene*
ROS: Reactive oxygen species
P5CSA: pyrroline-5-carboxylate synthase A
P5CSA: pyrroline-5-carbonlate synthase B
CAT: catalase
APX: Ascorbate peroxidase
ACS: *1-aminocyclo propane 1-carboxylic acid synthase*
SOSI: salt overly sensitive 1
NHX: sodium-hydrogen exchanger
HKT: high-affinity K^+^ transporter
ACS: *1-aminocyclo propnae 1-carbonxylic acid synthase*
CRISPR-Cas: Clustered regularly interspaced short palindromic repears)-Cas (CRISPR-associated) nucleases system
